# Long-term effect of food insecurity on body weight gain and metabolic risk in a context of high socioeconomic vulnerability in a medium-income country: the SANCuité cohort, Brazil, 2011–2022

**DOI:** 10.3389/fpubh.2025.1574499

**Published:** 2025-04-04

**Authors:** Jackson Silva Lima Laurentino, Isadora Macedo de Oliveira Martins-Costa, Rônisson Thomas de Oliveira-Silva, Ana Beatriz Macêdo Venâncio dos Santos, Poliana de Araújo Palmeira

**Affiliations:** ^1^Graduate Program in Science of Nutrition (PPGCN), Federal University of Paraíba, João Pessoa, Brazil; ^2^Nutrition and Public Health Studies and Research Group (Núcleo PENSO), Federal University of Campina Grande, Cuité, Brazil

**Keywords:** food insecurity, weight gain, obesity, social vulnerability, low socioeconomic status, cohort studies, Brazil

## Abstract

**Objective:**

Using longitudinal data from a study conducted in an area of high socioeconomic vulnerability in Brazil, we examined the long-term effects of food insecurity (FI) and social determinants on body weight gain (body weight, BMI) and metabolic risk (waist circumference - WC, waist-to-height ratio - WHtR) over 11 years of follow-up, conducted between 2011 and 2022.

**Methods:**

Face-to-face household interviews were conducted using the Brazilian Food Insecurity Scale to measure FI, and anthropometric measurements of weight, height, and WC were taken. Data analysis was performed in STATA 15.0 using multilevel mixed-effects regression with covariate adjustment and predicted marginal means with marginal differences.

**Results:**

Among the 210 individuals followed over 11 years, high prevalence of FI was observed (2011: 51.8%; 2022: 45.9%), central adiposity (2011: 83.8%; 2022: 88.6%), as well as a significant increase in the prevalence of high BMI (2011–2022: +16.7 *p* < 0.00), general obesity (2011–2022: +15.2 *p* < 0.00), and abdominal obesity (2011–2022: +0.5 *p* 0.02) over time. Multivariate analysis showed a positive association between BMI and body weight with mild, moderate, and severe FI after 8 and 11 years of follow-up among adults. A progressive increase in predicted body weight and BMI scores was observed among adults, with an increase of +5.6 (*p* 0.02) and + 2.3 (*p* 0.01) at the end of the follow-up, respectively, being higher in individuals with severe FI compared to those with food security. Among people ≥60 years old, WC and WHtR mean varied according to time and FI categories, with a positive association observed in mild and moderate FI, and an inverse association with severe FI at the end of the follow-up.

**Conclusion:**

FI is a risk factor for long-term weight gain and obesity, particularly in vulnerable populations, highlighting the need for intersectoral public policies to ensure food and nutrition security, combat obesity, and combat the structural causes of poverty and FI.

## 1 Introduction

The alarming rise in the prevalence of excess weight, including overweight and obesity, is a major global health crisis (1, 2). The population living with excess weight is concentrated mainly in low-and middle-income countries (LMICs) (3, 4). Environmental, social, nutritional, political, and commercial determinants of health and socioeconomic status contribute to the increased occurrence of excess weight in vulnerable populations in LMICs (5–7). Thus, people living in contexts of high socioeconomic vulnerability are at greater risk of excess weight and obesity (8, 9). Food insecurity (FI) is an economic and social condition characterized by limited or uncertain access to adequate food. FI is an indicator of socioeconomic vulnerability and inadequate access to adequate food (10). It remains a severe social and health problem worldwide, with a progressive rise in Latin America and the Caribbean, especially in South America (11–13).

FI is a potential risk factor for adult malnutrition (14, 15) and has been associated with obesity in many countries (14, 16–19), including Brazil (20–22). The association of FI-obesity is complex. This association is related, in particular, to malnutrition and the affordability of energy-dense foods (14, 23). The deprivation and difficulty in accessing adequate foods among people on FI lead to increased consumption of lower-cost foods with high caloric density, such as processed and ultra-processed foods (14, 23). These factors are intensified by social, economic, and regional inequalities (14), especially in LMICs (12). Poverty is a critical factor that contributes to FI and directly triggers the consumption of unhealthy diets. Consequently, FI and poverty contribute to the rise in the prevalence of excess weight and increased metabolic risk (12, 23, 24).

In Brazil, the Brazilian Institute of Geography and Statistics showed that 27.6% of Brazilian households (21.6 million Brazilians) were in FI in 2023, with 9.4% (7.4 million Brazilians) in moderate/severe FI (25). At the same time, according to the VIGITEL system, in 2023, 24.3% of adult Brazilians were living with obesity, with obesity rates being higher among adults with less education and in lower-income families, especially in the lower social classes (26).

Mitigating FI and obesity are targets of the Millennium Development Goals of the United Nations 2030 Agenda (27). Studies about the FI-obesity association have failed to explain the mediatory mechanisms of the relationship (14). The research cross-sectional models do not allow for exploring pathways and mechanisms underlying the relationship between FI and obesity, such as time of exposure to FI (14). Therefore, longitudinal studies are necessary.

In this article, using longitudinal data from an 11-year study conducted in an area of high socioeconomic vulnerability in Northeast Brazil, we examined the long-term effect of FI and social determinants on body weight gain and metabolic risk, using body weight, BMI, waist circumference, and waist-to-height ratio indicators in adults and people ≥60 years old.

## 2 Methods

### 2.1 Study design and population

We used data from the study “Effect of public policies on the food insecurity situation of families in the semi-arid northeast: cohort study, 2011–2022 (SANCuite cohort)” carried out by the Nutrition and Public Health Studies and Research Group (Núcleo PENSO), which monitored families in the municipality of Cuité, semi-arid region of the state of Paraíba, Brazil, for 11 years, between 2011 and 2022. The municipality selected for the research, Cuité, like approximately 70% of Brazilian cities; Cuité has approximately 20,000 inhabitants who face challenges related to water scarcity and the region’s low social and economic development (28). It is located in the semi-arid region of Northeast Brazil, in Paraíba, an area of extreme socioeconomic and climatic vulnerability. Due to these characteristics, the municipality was chosen to conduct the study.

**Figure 1:**
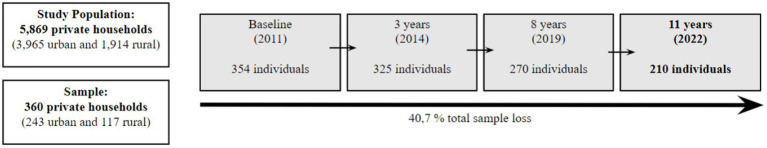
Flowchart of the sample of individuals from the SANCuite cohort, Cuité, Brazil, 11 years of follow-up (2011–2022).

The cohort began in 2011 (baseline), with a random and representative sample of the urban and rural population calculated based on an estimated prevalence of FI of 50%, a confidence interval of 95%, and a sampling error of 5%. Further details on the cohort baseline are available in Palmeira, Costa-Salles, and Perez-Escamilla (29). The follow-ups were performed in 2014, 2019, and 2022 via returns to the households of families surveyed at the baseline. The cohort consisted of adults (20–59 years old) and older people (≥ 60 years old). Pregnant women were excluded. At the end of data collection in 2022, 210 individuals were studied in all years of field research and comprised the sample of this study. The cohort of individuals had a sample loss of 40.7% (Figure 1).

### 2.2 Outcome variables

The primary outcome variables are body weight (kg), BMI (kg/m2), waist circumference (WC) (cm), waist-to-height ratio (WHtR), high BMI, general obesity, abdominal obesity risk, abdominal obesity, and central adiposity. Body weight, height, and WC were collected following WHO recommendations (30). All anthropometric measurements were performed in duplicate, and the mean of the two measurements was used for analysis.

High BMI (yes/no) was defined as the BMI of overweight or obesity (31). According to WHO recommendations (31), overweight and obesity were defined for adults as BMI between 25.0–29.9 kg/m2 and 30 kg/m2 and more, respectively; for people ≥60 years old, overweight was defined as BMI ≥ 27 kg/m2 (32) and obesity as ≥30 kg/m2 (31). General obesity (yes/no) was defined as a BMI of 30 kg/m2 and more (31). Abdominal obesity risk was defined according to cutoff points for WC as Low risk (WC < 94 for men and < 80 for women) and High risk (WC ≥ 94 for men and ≥ 80 for women) (33). Abdominal obesity (yes/no) was defined as WC ≥ 102 cm for men and ≥ 88 cm for women [33 Central adiposity (yes/no) was a WHtR >0.5 (34, 35)]. We used WC, WHtR, abdominal obesity risk, abdominal obesity, and central adiposity as proxy for metabolic risk.

The Multiple imputation technique imputed 11.4% (n = 24) of missing weight, WC, and BMI data. WC data collection began in the first follow-up of the cohort (2014) and was not collected at baseline; Thus, missing WC data (n = 210) at baseline (2011) were also imputed. Time, height, sex, and age were control variables in the imputation model for body weight and BMI, while time, sex, and age were used for WC.

### 2.3 Exposure variable

The FI level was the independent variable used in the present study. This variable was measured using the Brazilian Food Insecurity Scale (EBIA), a method validated in Brazil in 2003 to assess household FI based on the dimension of access to food in the Brazilian population (36). The EBIA is a household experience-based scale adapted from the US Household Food Security Survey Module (36). We classified the household FI of individuals according to the sum of affirmative responses to EBIA items and household composition (36): Food security (FS) (score = 0); Mild FI (score = 1–5 in households with children/adolescents, 1–3 in adult-only households) Moderate FI (score = 6–9 in households with children/adolescents, 4–5 in adult-only households); Severe FI (score = 10–14 in households with children/adolescents, 6–8 in adult-only households).

### 2.4 Socio-demographic characteristics

The individuals were classified by sex (male or female), age group (adults or people ≥60 years old), skin color/race (white or black/mixed-race), high schooling (high school or more), and low schooling (up to elementary school). The households of the individuals were also characterized by housing area (urban or rural), access to cash transfer programs (Bolsa Família 2011–2021 and Auxílio Brasil 2021–2022), and monthly per capita income plus the value of cash transfer programs (above/below ½ the minimum wage). The Brazilian minimum wage was: 2011, R$ 545 ($US 340.6); 2014, R$ 724 ($US 308,1); 2019, R$ 998 ($US 253.3); and 2022, R$1212 ($US 253.3). We also use the “time” variable. The “time” variable describes the years of follow-up, which are: baseline (2011), 3 years (2014), 8 years (2019), and 11 years (2022). These covariates were used for the analyses and defined based on studies about the FI and the FI-obesity association (22, 37–39).

### 2.5 Statistical analysis

All statistical analyses were conducted using Stata 15.0 (40). Continuous variables are presented as means and categorical variables as proportions. Simple and average frequencies were estimated for each year of follow-up in the study: baseline (2011), 3 years (2014), 8 years (2019), and 11 years (2022). We applied the Cochran Q test to assess significant changes over time for category variables. Repeated Measures Analysis of Variance (ANOVA) was used to analyze how the means of the outcome variables vary over the follow-up period within each age group. The model analyzed was as follows: “by Age group, sort: ANOVA outcome-variable id time, repeated(time).” Mixed-effects Analysis was used to investigate how FI and time variables, both in their direct effects and interactions, influence the outcome variables.

We conducted a Multilevel Mixed-effects Regression of Body weight, BMI, WC, and WHtR with FI over time, adjusted by sex, income, and an interaction term between FI, age group, and time (FI#age#time). Next, the predicted marginal means for the outcome variables were estimated based on the time and FI variables, stratified by age group (adults and people ≥60 years old). The predicted means for each outcome were plotted separately. Differences between the means obtained over time were estimated, as well as comparisons between mild, moderate, and severe FI with the food security (FS) category, using the contrast command, and this allowed for the assessment of the effect of each level of FI and time on the outcome variables.

All multivariate models were estimated with robust variance adjustment and by considering unstructured covariance when tested, and the models did not exhibit collinearity. The sample size of this study can detect a true odds ratio of at least 2.42 with a 95% confidence level and 80% power (1-beta). Results were reported as significant with a *p* < 0.05.

## 3 Results

The studied population showed a higher prevalence of females (87.6%), black/mixed-race individuals (70.0%), and urban residents (71.4%, 2011–2014: +2.9%). The proportion of people ≥60 years old, with higher education and income above ½ minimum wage, increased over the follow-up period. FI was also observed to persist over time, with a decrease from baseline (2011: 51.8%) during the first 8 years of follow-up (2019: 34.8%) and a subsequent increase in the final year (2022: 45.9%) (Table 1).

**Table 1:** Socioeconomic characteristics and food insecurity status in individuals over time, SANCuité cohort, Brazil, 11 years of follow-up (840 observations).

Variables	Baseline (2011) % (n)	3 years (2014) % (n)	8 years (2019) % (n)	11 years (2022) % (n)	△ (2011–2022)	p*
≥ 60 years	16.6 (35)	21.9 (46)	32.4 (68)	37.6 (79)	+21	**<0.001**
Urban area	71.4 (150)	71.9 (151)	73.8 (155)	74.3 (156)	+2.9	-
Above ½ minimum wage^1^	63.8 (134)	70.9 (149)	77.1 (162)	86.2 (181)	+22.4	**<0.001**
Education^2^						
High schooling	30.5 (64)	31.4 (66)	32.8 (69)	38.1 (80)	+7.6	**<0.001**
Low schooling	69.5 (146)	68.5 (144)	67.1 (141)	61.9 (130)	−7.6	
Food insecurity (FI)						
Food security	48.1 (101)	62.4 (131)	65.2 (137)	54.1 (113)	+6	**<0.001**
Mild FI	27.6 (58)	20.9 (44)	19.5 (41)	30.1 (63)	+2.5	
Moderate FI	14.3 (30)	8.1 (17)	10.0 (21)	11.0 (23)	−3.3	
Severe FI	10.0 (21)	8.6 (18)	5.2 (11)	4.8 (10)	−5.2	

*Q Cochran test; ^1^Monthly family income per capita plus the value of cash transfer programs; ^2^High schooling (high school or more), low schooling (up to elementary school).

Bold text indicates statistical significance at *p*-value < 0.001.

### 3.1 Changes in body weight, BMI, and metabolic risk over time

**Figure 2:**
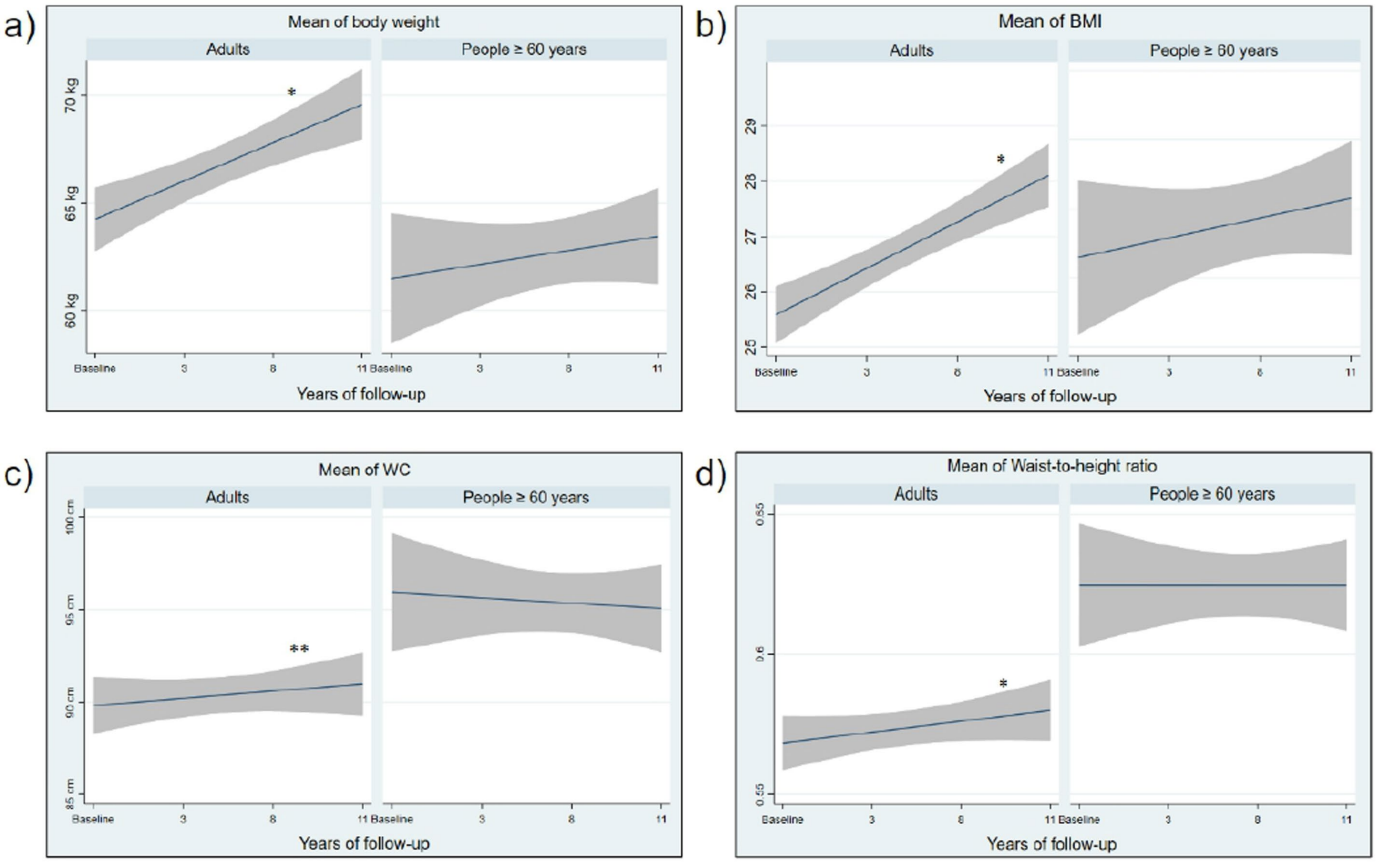
Means of body weight, BMI and metabolic risk from 11 years of follow-up, by age groups. **(A)** Body weight; **(B)** Body mass index (BMI); **(C)** Waist circumference (WC); **(D)** Waist-to-height ratio. *p* calculated by repeated measures ANOVA. *p < 0.000. **p < 0.05.

In Figure 2, the means of body weight, BMI, WC, and WHtR were plotted according to the years of follow-up, showing a significant increase in these scores over time in the adult group. A progressive increase in the prevalence of high BMI (+16.7%, *p* < 0.00) and general obesity (+15.2%, *p* < 0.00) was observed over time. A prevalence of over 70% of high abdominal obesity risk was observed in the population, with these rates being maintained throughout the follow-up period. The prevalence of abdominal obesity showed fluctuations in proportions, with an increase from baseline (53.3%) to after 8 years of follow-up (61.4%), followed by a reduction in the last follow-up (2022: 53.8%, *p* = 0.02). A prevalence of over 80% of central adiposity was also observed in the population, with these rates being maintained over time (Table 2).

**Table 2:** Prevalence of high BMI, obesity and metabolic risk in individuals over time, SANCuité cohort, Brazil, 11 years of follow-up (840 observations).

Variables	Baseline % (n)	3 years % (n)	8 years % (n)	11 years % (n)	△ (2011–2022)	p*
High BMI						
No	51.9 (109)	48.6 (102)	41.9 (88)	35.2 (74)	−16.7	**<0.00**
Yes	48.1 (101)	51.4 (108)	58.1 (122)	64.8 (136)	+16.7	
General obesity						
No	83.8 (176)	84.3 (177)	74.3 (156)	68.6 (144)	−15.2	**<0.00**
Yes	16.2 (34)	15.7 (33)	25.7 (54)	31.4 (66)	+15.2	
Abdominal obesity risk						
Low risk	23.8 (50)	23.3 (49)	18.1 (38)	21.9 (46)	−1.9	0.33
High risk	76.2 (160)	76.7 (161)	81.9 (172)	78.1 (164)	+1.9	
Abdominal obesity						
No	46.7 (98)	50.9 (107)	38.6 (81)	46.2 (97)	−0.5	**0.02**
Yes	53.3 (112)	49.1 (103)	61.4 (129)	53.8 (113)	+0.5	
Central adiposity						
No	16.2 (34)	16.2 (34)	10.5 (22)	11.4 (24)	−4.8	0.08
Yes	83.8 (176)	83.8 (176)	89.5 (188)	88.6 (186)	+4.8	

*Q Cochran test.

Bold text indicates statistical significance at *p*-value < 0.05.

### 3.2 Association between FI and body weight, BMI, and metabolic risk

When comparing the means of body weight, BMI, WC, and WHtR according to FI categories, we found an association of Mild FI with body weight (*p* = 0.012) and BMI (*p* = 0.009) after 8 years of follow-up and an association of Severe FI with BMI after 8 years (*p* = 0.039) and 11 years of follow-up (*p* = 0.021), when compared to the reference group (FS at baseline). The means of WC and WHtR did not show an association with FI (Supplementary Table 1).

**Figure 3:**
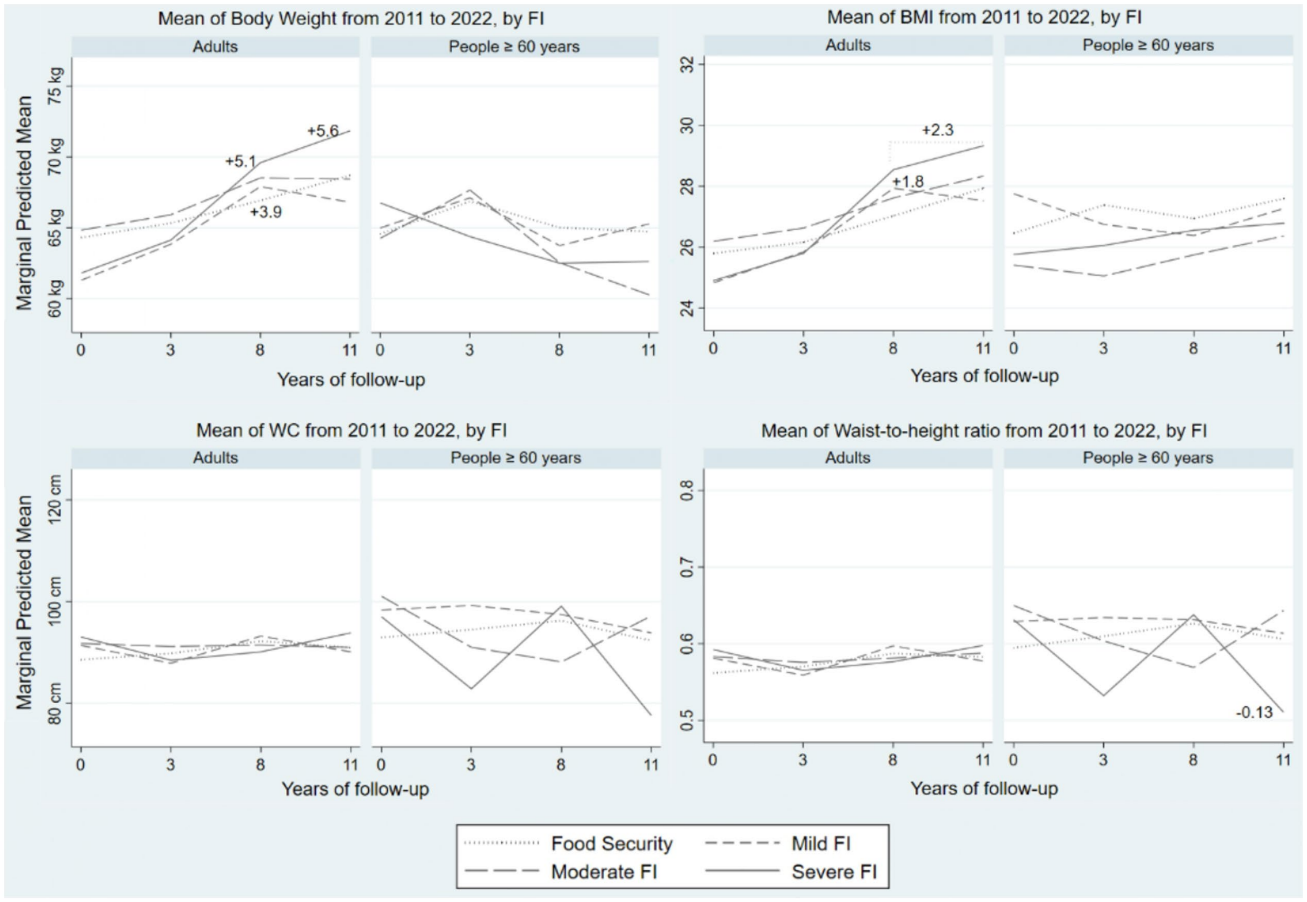
Change in the predicted mean of BMI, body weight and metabolic risk over time according to food insecurity, by age groups: Cuité, Brazil, 11 years of follow-up. Mixed-effects logistic regression, margms predicted by age groups and contrast of predicted margins. Significant contrast results are shown (*p* < 0.05).

Table 3 presents the results of the multivariate mixed-effects analysis of the association between outcome variables and FI, including an interaction term with time by age group. Figure 3 displays the predicted means for body weight, BMI, WC, and WHtR over time by age group, considering the results of the association *contrast*.

**Table 3:** Multilevel mixed-effects regression of body weight, WC, waist-to-height, and body max index with food insecurity over time, SANCuité cohort, Brazil, 2011–2022 (840 observations).

Variables	Body weight		BMI		WC		Waist-to-height ratio	
	*β*	*p*	*β*	*p*	*β*	*p*	*β*	*p*
Food Security x Baseline	Ref.		Ref.		Ref.		Ref.	
Adults								
Mild FI # 3 years	−0.46	0.620	0 0.04	0.913	0.69	0.749	−0.00	0.834
Mild FI # 8 years	3.59	**0.000**	2.13	**0.000**	4.64	**0.035**	0.03	**0.013**
Mild FI # 11 years	2.49	**0.042**	1.72	**0.001**	1.53	0.496	0.01	0.276
Moderate FI # 3 years	1.61	0.252	0.83	0.181	2.59	0.345	0.01	0.444
Moderate FI # 8 years	4.21	**0.000**	1.82	**0.001**	2.90	0.265	0.01	0.241
Moderate FI # 11 years	4.13	**0.001**	2.53	**0.000**	2.42	0.291	0.02	0.079
Severe FI # 3 years	−0.17	0.894	0.00	0.993	−0.10	0.969	0.00	0.823
Severe FI # 8 years	5.28	**0.001**	2.74	**0.004**	1.54	0.652	0.01	0.500
Severe FI # 11 years	7.53	**0.002**	3.53	**0.000**	5.27	**0.020**	0.03	**0.015**
People ≥ 60 years								
Mild FI # 3 years	3.08	0.062	1.06	0.181	10.3	**0.000**	0.07	**0.000**
Mild FI # 8 years	−0.28	0.897	0.70	0.540	8.55	**0.000**	0.06	**0.000**
Mild FI # 11 years	1.25	0.502	1.59	**0.007**	4.94	**0.038**	0.05	**0.001**
Moderate FI # 3 years	3.64	**0.001**	−0.62	0.215	2.11	0.389	0.04	**0.021**
Moderate FI # 8 years	−1.48	0.433	0.06	0.951	−0.75	0.875	0.00	0.861
Moderate FI # 11 years	−3.75	**0.001**	0.68	0.120	8.29	**0.000**	0.07	**0.000**
Severe FI # 3 years	0.35	0.786	0.37	0.496	−6.04	**0.002**	−0.03	**0.015**
Severe FI # 8 years	−1.52	0.333	0.87	0.458	10.2	**0.000**	0.07	**0.000**
Severe FI # 11 years	−1.40	0.531	1.10	0.090	−11.3	**0.000**	−0.005	**0.000**

Baseline (2011); 3 years (2014); 8 years (2019); 11 years (2022).

BMI, body mass index; FI, food insecurity; WC, waist circumference; *β*, *β* coefficient.

Bold text indicates statistical significance at *p*-value < 0.05 or < 0.001.

For body weight, a positive association was observed with all categories of FI in the later years of follow-up among adults. In contrast, for people ≥60 years old, this association was observed only with Moderate FI (Table 3). The predicted means for body weight in adults were higher in the Mild FI category (+3.9 kg at 8 years of follow-up, *p* 0.005) when compared to the Food security category. In the Severe FI category, this difference was even more significant and progressively increased in the later years of follow-up (+5.1 kg to 8 years, *p* 0.007, and + 5.6 kg to 11 years, *p* 0.02).

For BMI, the data also showed a positive association with all categories of FI in the later years of follow-up among adults. For people ≥60 years old, this association was observed only in the Mild FI category (Table 3). The predicted means for BMI in adults were 1.8 units higher in the Mild FI category (p 0.002) and 2.3 units higher in the Severe FI category (p 0.01) after 8 years of follow-up, compared to the Food Security category (Figure 3).

A positive association between WC and Mild and Severe FI was observed in the later years of follow-up among adults. In contrast, for people ≥60 years old, this association was observed with all categories of FI in the later years of follow-up (Table 3). The predicted means for WC showed no differences. A positive association between WHtR and Mild and Severe FI was observed in the later years of follow-up among adults. For people ≥60 years old, an association between WHtR and all categories of FI was found in the later years of follow-up (Table 3). The predicted means for WHtR in people ≥60 years old were lower in the Severe FI category (−0.13, p 0.04) compared to the Food Security category after 11 years of follow-up. Notably, all predicted means for WHtR were above 0.5, the cutoff point indicating central adiposity (Figure 3).

All these variations in means had their statistical significance confirmed by *contrasts* of predictive margins. Thus, our results indicate that, among adults in a state of FI, the predicted means for body weight and BMI progressively increased over time and with the severity of FI (Figure 3).

## 4 Discussion

### 4.1 Key findings

The results of this study, conducted with a population exposed to vulnerabilities and deprivation over 11 years in the semi-arid region of one LMIC, revealed weight gain and increases in average BMI scores, leading to a rise in the prevalence of obesity and the persistence of high metabolic risk and central adiposity in the studied population over time. Long-term exposure to mild and severe FI was associated with increased BMI and body weight in the adult population but not among people ≥60 years old.

### 4.2 Long-term effects of food insecurity on body weight gain and metabolic risk

The long-term effect of FI is a significant risk factor for body weight gain and increased metabolic risk. It serves as an essential determinant for the development of obesity, as observed in this longitudinal cohort study. Previous cross-sectional studies have reported an association between FI and adult obesity (16, 17, 41, 42). In the context of LMICs, longitudinal studies about the long-term effects of FI on obesity are scarce yet, especially in adults. Nevertheless, evidence from cross-sectional studies conducted in Mexico (18, 43), Brazil (20, 21, 44), Ethiopia (45), and Iran (19), suggested that individuals facing FI are more likely to develop overweight and obesity. This is particularly evident in regions and populations with low socioeconomic status and food access disparities.

In the US, longitudinal studies have demonstrated a positive association of household FI (46), high cumulative social risk (including FI) (47), and economic adversity (income, FI, debt, and housing insecurity) (48) with increases in BMI scores over time in children. On the other hand, in a context where the population involved in the research received financial support from government agencies to cope with FI, Huang et al. found no association between increased BMI and FI in economically vulnerable adolescents in Taiwan. In this case, the possible improvement in food security status blocked the impact on the increase in BMI over the time of the study (49).

In US adults, Lohman et al. found a positive association between FI during adolescence and elevated BMI 16 years later in women (50). In a prospective cohort matched for age, sex, race/ethnicity, and BMI, Cheung et al. found that baseline FI was associated with more significant BMI gains after 3.2 years of follow-up in adults attending a community health center in Massachusetts (51). In a longitudinal study conducted in Minnesota, Hooper et al. demonstrated that individuals who lived in households with FI during adolescence had higher elevated BMI scores after 8 years of follow-up compared to those in households with food security and suggested that a household FI experienced during adolescence predisposes to the incidence of elevated BMI in adulthood (52).

Concerning this, the impacts of FI on body weight gain and metabolic risk depend on factors such as the duration and severity of exposure to FI and the socioeconomic and environmental context. Body weight gain and metabolic risk are highly associated with physical activity. Since FI is a condition of social vulnerability, it is important to raise the hypothesis that in families living with moderate and severe FI, regular physical activity may not be a priority when there is food deprivation. National data on the practice of physical activity in the capitals of Brazil showed that 37% of the individuals in the population studied did not achieve a sufficient level of physical activity, and it is important to highlight that physical activity decreases as the level of education decreases (26). Data from a systematic review of Brazilian studies on FI-Obesity raised the hypothesis of an association between FI and Obesity and NCDs precisely because of the presence of a sedentary lifestyle in contexts of social vulnerability (22).

Another factor highly associated with obesity is stress. Physiological responses to chronic stress can increase cortisol levels and influence body fat distribution, incredibly visceral adipose tissue. Excessive visceral fat accumulation in the abdominal region increases the risk of adverse health outcomes, such as obesity, cardiovascular disease, and diabetes (4, 8). Thus, prolonged stress associated with persistent FI can lead to changes in metabolic functioning, promoting abdominal fat accumulation. In addition, FI can impact the intestinal microbiota, which can also contribute to weight gain (4, 8, 53).

Over time, families exposed to prolonged FI develop and resort to strategies to ensure survival and access to food, consequently managing persistent FI. These strategies result in lower consumption of fresh and minimally processed foods and increased consumption of processed and ultra-processed foods (16, 53, 54); these food-related decisions are primarily based on the cost of foods (55). As a result, these families consume diets of low nutritional quality and with reduced dietary diversity (54, 56). Furthermore, the dynamics of FI experience over time, with prolonged periods of food deprivation and unpredictable eating patterns, contribute to the emergence of stress states that promote the development of obesity (57–59). These factors, combined with the chronic stress of living with food deprivation (60–62), may explain the tendency for individuals experiencing FI to gain weight over time. Thus, the dynamics of FI over time lead to the chronicity of exposure to risk factors associated with living in FI, contributing to the long-term effect of FI in the development of obesity and increased metabolic risk.

Therefore, our findings reveal insights into the long-term effects of FI on weight gain and metabolic risk and suggest mechanisms that may play a role in the association between FI and obesity.

### 4.3 Implications

This study has important implications for future weight management interventions in individuals in the context of FI and social inequalities. Obesity within the context of FI adds a layer of complexity to the current obesity epidemic (14). Thus, the long-term effects of FI on the risks of developing obesity among FI families should be considered. Therefore, the challenge of FI-obesity needs intersectoral care approaches. Health promotion, prevention, and treatment actions for overweight and obesity must be supported by intersectoral actions and policies to promote food and nutritional security and reduce social inequalities.

To face obesity in the context of FI and social inequalities involves considering a food system-level approach that efficiently addresses systemic and environmental determinants (63). The food environment provides the context for food choices; therefore, system changes are necessary to ensure that families can afford foods that promote a healthy diet. Concerning this issue, one of the primary objectives of facing obesity is to increase the consumption of fresh and minimally processed foods while reducing the consumption of processed and ultra-processed foods within the population. Achieving this requires coordinated actions and policies across agriculture, social assistance, and the economy to ensure the availability and accessibility of fresh and minimally processed foods for the population.

Brazil has a network of policies, programs, and actions aimed at reducing FI and obesity. Some examples include the National Food and Nutrition Policy (PNAN), which addresses obesity as both a risk factor and a disease and proposes improvements in food, nutrition, and health conditions, as well as ensuring the food and nutrition security of the Brazilian population (64), and the National Food and Nutrition Security Policy (PNSAN), an intersectoral policy with the perspective of adequate and healthy food, which treats obesity as a social problem and FI and proposes new ways to produce, market, and consume food to modify eating practices (65).

In this regard, a prominent program is the National School Feeding Program (PNAE), which provides free daily meals to students in Brazilian public schools. The meal preparation guidelines prioritize consuming fresh or minimally processed foods, purchasing products from family farming, and restricting ultra-processed foods (limited to 15%) (66). In addition, Brazil has invested in a set of guidance materials for the population and public managers, such as the Dietary Guidelines for the Brazilian population (67) and the Reference Framework for Food and Nutrition Education for Public Policies (68), as well as measures that promote access to adequate and healthy food, and consequently, the reduction of FI and obesity. These include the decree on the composition of the new Basic Food Basket and the reduction of taxes on products from the new Basic Food Basket, which excludes ultra-processed foods (69, 70).

Evidence shows that the food environment mediates the association between FI-obesity (51, 71), especially the accessibility of fresh produce in the neighborhood (72). Another critical aspect of the food environment concerns the places where people in FI purchase food. In Brazil, nationally, most food groups are predominantly purchased at supermarkets. Nonetheless, households experiencing moderate or severe FI rely more on small markets for essential items such as beans, rice, proteins, and ultra-processed foods. These findings highlight the need for public policies to improve vulnerable populations’ food access (73).

Therefore, policy efforts to mitigate obesity among families with FI should focus on the availability and affordability of neighborhood fresh produce and the social environment in which communities are embedded. Income access, stability, and food costs are key determinants of the FI-obesity relationship (62). Findings indicate that individuals facing FI-obesity food decisions are primarily based on food costs and influenced by family income levels, highlighting income as a barrier to healthy eating (51, 55). In this context, income redistribution policies and reducing prices for fresh and minimally processed foods are necessary.

Furthermore, evidence shows that FI reduces the effectiveness of interventions and treatments to reduce overweight and obesity (74). Thus, the findings of this study are essential for shaping care pathways for individuals living with overweight and obesity. The development of these care pathways should consider the effects of FI on the development of overweight and obesity. It should be grounded in an intersectoral approach, encompassing the sectors of social assistance, education, economy, and agriculture. In this sense, food and nutritional surveillance is crucial to face FI-obesity. Screening for FI has the potential to identify individuals and families at risk of increased BMI, as it serves as an indicator of inadequate access to proper alimentation and the risk of obesity, thereby enabling their inclusion in intersectoral care pathways.

By demonstrating the long-term effect of FI on increased body weight and BMI in a population experiencing socioeconomic vulnerability, this study highlights the need for a sustained and targeted set of intersectoral public policies to combat hunger, promote health, and reduce obesity in vulnerable areas.

### 4.4 Strengths and limitations

We acknowledge that the limitations of this study may include the absence of predictive and confounding variables, such as physical activity, stress, sleep quality, blood pressure, lipid profile, and related biochemical markers associated with obesity. In our study, it was impossible to collect these variables due to logistical data collection issues in remote regions and the availability of financial resources. However, we emphasize that weight, height, and circumference measurements have been widely used in population studies precisely because of the feasibility of implementation in data collection, which justified the choice of these variables. Future longitudinal studies should consider these variables when examining and controlling the analysis of the FI-obesity association.

However, this is the first study to analyze the FI-obesity association longitudinally in a population of one LMIC. The study’s strengths include its longitudinal approach and the 11-year follow-up period, which allows for an in-depth analysis of changes over time, which helps to understand the long-term effects of FI on obesity. Another strength concerns the geographic and sociodemographic context in which the study was conducted, an area of high socioeconomic vulnerability in Northeastern Brazil. Finally, this study helps to address significant scientific gaps regarding the FI-obesity association, particularly in terms of its temporal dynamics. It may contribute to the development of public policies to mitigate this issue.

## 5 Conclusion

Food insecurity is a risk factor for weight gain and increased obesity and metabolic risk over time, particularly in populations in contexts of socioeconomic vulnerability. The long-term effects of FI significantly impact weight gain, eating behaviors, and psychosocial and metabolic factors. Therefore, this issue must be faced through intersectoral public policies focused on ensuring food and nutrition security and combating obesity. Furthermore, such policies must address the structural causes of poverty and FI to promote long-term health. Longitudinal cohort study can attest to the findings of the current study.

## Data Availability

The raw data supporting the conclusions of this article will be made available by the authors, without undue reservation.
