# Supplementary material for: Long-term effect of food insecurity on body weight gain and metabolic risk in a context of high socioeconomic vulnerability in a medium-income country: the SANCuité cohort, Brazil, 2011–2022
Source: Front Public Health. 2025 Apr 4;13:1574499. doi: 10.3389/fpubh.2025.1574499 (PMC12006142; doi:10.3389/fpubh.2025.1574499)
Supplement: Supplementary file 1 [file Table_1.pdf]

Supplementary Material

Supplementary Table 1. Mean of body weight, waist circumference, waist-to-height ratio, and BMI by food insecurity status over time, SANCuité cohort, Brazil, 11 years of follow-up (840 observations).

**Food insecurity**	**Body weight (Kg)**<br>Mean (CI%95)				**Waist Circumference (cm)**<br>Mean (CI%95)				**Waist-to-height ratio**<br>Mean (CI%95)				**BMI (Kg/m^2)**<br>Mean (CI%95)			
	**Baseline**	**3 years**	**8 years**	**11 years**	**Baseline**	**3 years**	**8 years**	**11 years**	**Baseline**	**3 years**	**8 years**	**11 years**	**Baseline**	**3 years**	**8 years**	**11 years**
Food Security	64.8<br>(62.6~67.1)	65.5<br>(63.4~67.6)	65.9<br>(63.9~68.0)	66.9<br>(65.5~69.3)	89.5<br>(87.1~91.9)	91.0<br>(88.9~93.2)	94.0<br>(91.9~96.0)	92.0<br>(89.8~94.3)	0.56<br>(0.55~0.58)	0.58<br>(0.56~0.59)	0.60<br>(58.0~0.61)	0.59<br>(0.57~0.60)	26.0<br>(25.3~26.8)	26.4<br>(25.7~27.1)	26.9<br>(26.1~27.6)	27.6<br>(26.8~28.4)
Mild FI	62.6<br>(59.8~65.4)	64.0<br>(60.9~67.1)	**67.3**<br>**(63.9~70.8)**	67.1<br>(64.0~70.3)	92.2<br>(88.5~96.0)	89.1<br>(85.8~92.3)	93.5<br>(89.6~97.3)	91.2<br>(88.2~94.1)	0.58<br>(0.56~0.61)	0.56<br>(0.54~0.58)	0.60<br>(0.57~0.62)	0.58<br>(0.56~0.60)	25.5<br>(24.3~26.6)	25.7<br>(24.6~26.8)	**28.0**<br>**(26.7~29.4)**	27.7<br>(26.6~28.8)
Moderate FI	64.5<br>(61.0~67.9)	70.2<br>(64.5~75.9)	68.6<br>(62.9~74.3)	65.4<br>(60.6~70.2)	92.7<br>(88.0~97.5)	93.4<br>(88.0~99.0)	91.5<br>(86.1~96.8)	93.7<br>(89.3~98.1)	0.59<br>(0.56~0.62)	0.59<br>(0.55~0.62)	0.58<br>(0.54~0.62)	0.61<br>(0.58~0.64)	26.2<br>(24.8~27.7)	27.9<br>(25.7~30.0)	27.8<br>(25.6~30.0)	27.7<br>(26.2~29.3)
Severe FI	58.7<br>(54.0~63.5)	62.8<br>(57.8~67.8)	68.2<br>(62.4~74.0)	70.2<br>(61.8~78.7)	92.0<br>(85.8~98.2)	87.0<br>(82.3~91.7)	92.6<br>(85.8~99.5)	89.4<br>(82.5~96.4)	0.59<br>(0.55~0.63)	0.56<br>(0.53~0.59)	0.58<br>(0.54~0.63)	0.57<br>(0.53~0.61)	24.3<br>(23.1~25.5)	26.2<br>(24.6~27.8)	**27.6**<br>**(25.4~29.8)**	**28.7**<br>**(25.7~31.7)**
